# Alpha-tocopherol transfer protein disruption confers resistance to malarial infection in mice

**DOI:** 10.1186/1475-2875-9-101

**Published:** 2010-04-19

**Authors:** Maria S Herbas, Yoshiko Y Ueta, Chie Ichikawa, Mayumi Chiba, Kana Ishibashi, Mototada Shichiri, Shinya Fukumoto, Naoaki Yokoyama, Motohiro Takeya, Xuenan Xuan, Hiroyuki Arai, Hiroshi Suzuki

**Affiliations:** 1 Research Unit for Functional Genomics, National Research Center for Protozoan Diseases, Obihiro University of Agriculture and Veterinary Medicine, Nishi 2-13, Inada, Obihiro, 080-8555 Japan; 2 Health Technology Research Center, National Institute of Advanced Industrial Science and Technology, Ikeda, Osaka, 563 Japan; 3 Department of Cell Pathology, Graduate School of Medical Science, Kumamoto University, 1-1-1, Honjo, Kumamoto, 860-8556 Japan; 4 Graduate School of Pharmaceutical Science, The University of Tokyo, 7-3-1, Hongo, Bunkyo-ku, Tokyo, 113-0033 Japan; 5 The United Graduate School of Veterinary Sciences, Gifu University, 1-1 Yanagido, Gifu City, 501-1193 Japan

## Abstract

**Background:**

Various factors impact the severity of malaria, including the nutritional status of the host. Vitamin E, an intra and extracellular anti-oxidant, is one such nutrient whose absence was shown previously to negatively affect *Plasmodium *development. However, mechanisms of this *Plasmodium *inhibition, in addition to means by which to exploit this finding as a therapeutic strategy, remain unclear.

**Methods:**

α-TTP knockout mice were infected with *Plasmodium berghei *NK65 or *Plasmodium yoelii *XL-17, parasitaemia, survival rate were monitored. In one part of the experiments mice were fed with a supplemented diet of vitamin E and then infected. In addition, parasite DNA damage was monitored by means of comet assay and 8-OHdG test. Moreover, infected mice were treated with chloroquine and parasitaemia and survival rate were monitored.

**Results:**

Inhibition of α-tocopherol transfer protein (α-TTP), a determinant of vitamin E concentration in circulation, confers resistance to malarial infection as a result of oxidative damage to the parasites. Furthermore, in combination with the anti-malarial drug chloroquine results were even more dramatic.

**Conclusion:**

Considering that these knockout mice lack observable negative impacts typical of vitamin E deficiency, these results suggest that inhibition of α-TTP activity in the liver may be a useful strategy in the prevention and treatment of malaria infection. Moreover, a combined strategy of α-TTP inhibition and chloroquine treatment might be effective against drug resistant parasites.

## Background

Despite recent advances in understanding malaria and *Plasmodium*, the parasite responsible for the disease, 500 million cases of clinical malarial in over 100 countries still occur. This disease poses a public health problem for 3.3 billion people, a number representing a staggering 50% of the world's population. Furthermore, the global death figure for malaria reaches more than 1 million each year [1]. A number of factors affect the severity of malaria, including the size of the sporozoite infective dose, host nutritional status, acquired immunity level, host genetic factors, parasite features and even certain associated socioeconomic factors [2-7]. Although micronutrient malnutrition is usually highly prevalent in areas in which malaria is endemic, the contribution of these micronutrient deficiencies to malarial symptoms is often overlooked.

Vitamin E is a powerful anti-oxidant that acts mainly in the lipid phase of cells and has a primary role in preventing the oxidation of polyunsaturated fatty acids [8]. While vitamin E deficiency seems to have both protective and adverse effects in malarial infection, the involvement of vitamin E in the genesis of malarial illness is still controversial [9]. The clinical observations that feeding famine victims with grain exacerbated the effects of cerebral malaria were attributed to the vitamin E content of the grain that subsequently influenced severity of malaria symptoms [10]. In addition, according to the results of animal studies, dietary vitamin E deficiency is thought to protect against malarial infection, presumably because the absence of this anti-oxidant leads to an increase in oxygen radicals production derived from the immune response of the host against the infection, consequently making an inhospitable environment for the parasite [11,12]. However, even if it were shown to be possible to utilize vitamin E deficiency for the prevention or treatment of malaria, it would be quite difficult to actually lower vitamin E in circulation via nutritional manipulation because the majority of daily foods in a normal diet contain significant amounts of vitamin E [8].

Vitamin E is transported in plasma lipoproteins and, unlike other fat-soluble vitamins, has no specific plasma carrier protein, however, alpha-tocopherol transfer protein (α-TTP), a liver cytosolic protein, acts as an important regulator of vitamin E concentration in circulation [13,14]. It does this through binding specifically α-tocopherol amongst the other tocopherols, including β and γ-tocopherol, in the liver. Targeted disruption of the *α-TTP *gene revealed that α-tocopherol concentration in circulation was regulated by *α-TTP *[13,15]; heterozygous mutant mice contained plasma concentrations of α-tocopherol half that found in wild type mice while homozygous mutants were shown to have undetectable levels of α-tocopherol in circulation [14]. Actual mechanism is not known. However, it is postulated that chylomicrons remnants with extra amount of α-tocopherol leaked in to the circulation.

The ability to manipulate α-tocopherol levels, and consequently vitamin E levels, via *α-TTP *gene inhibition inspired us to revisit the impact of serum vitamin E levels on the severity of malarial infection using an established rodent malarial model. Thus, *α-TTP *inhibition was examined as a potential application for the prevention or treatment of malarial infection. The results indicated that *α-TTP *inhibition led to parasite DNA damage sufficient to inhibit proliferation. Moreover, a combined therapy with chloroquine (CQ) seems to be useful as a new strategy for the treatment of malaria.

## Methods

### Animals and malaria infection

Since C57BL/6J mice are frequently used as an experimental model in biomedical sciences, α-TTP knockout mice were generated with a C57BL/6J background [14]. These knockout mice were bred in our own colony. BALB/c mice were known to be murine malaria resistant strain. Adult α-TTP knockout mice with a C57BL/6J genetic background, C57BL/6J and BALB/c mice were infected with 4 × 10^5 ^*Plasmodium berghei *NK65 or 4 × 10^4 ^*Plasmodium yoelii *17XL infected red blood cells (IRBCs) by intraperitoneal injection, and their survival was monitored. Six animals were used in each experimental group. Mice were fed with a commercial diet (CE-2, containing 45 mg/kg of D-α-tocopherol, CLEA Japan, Tokyo, Japan) or α-tocopherol supplementation (CE-2 with supplementary D-α-tocopherol, 600 mg/kg, CLEA Japan, Tokyo, Japan). Furthermore, to determine the effect of vitamin E deficiency on the virulence of *P. berghei *NK65, α-TTP knockout or C57BL/6J mice were inoculated with *P. berghei *NK65 (4 × 10^5 ^IRBCs) recovered from α-TTP knockout or wild type mice at day 9 post-infection, and their survival was monitored. In a part of the experiments, mice infected with *P. berghei *NK65 were administered 0-7.5 mg/kg of CQ (C6628, Sigma-Aldrich, St. Louis, USA) on day 0, 1 and 2 after infection, and their survival was monitored. All experiments described in the present study were conducted in accordance with the Guiding Principles for the Care and Use of Research Animals of the Obihiro University of Agriculture and Veterinary Medicine, Japan.

### Haematological analysis

Haematological parameters, such as the number of red blood cells (RBCs), white blood cells (WBCs), haemoglobin concentration, and haematocrit were determined with an autohaematology analyzer (Celltac α, MEK-6358, Nihon Kohden, Tokyo, Japan) during the course of infection. Six mice were used for each experimental group. For the reticulocyte count, 2 μl of whole blood taken from the tail vein were mixed with 2 μl of Brilliant Crystal Blue (Wako, Tokyo, Japan). Then thin smears of sample were prepared, dried at room temperature and stained with Giemsa (Merck, Darmstadt, Germany). The numbers of infected and uninfected reticulocytes, as well as mature RBCs, were determined under light microscopy.

### The comet assay

DNA damage of the parasites infecting the mice was assessed by single-cell gel electrophoresis (comet assay) [16]. Evaluation of the shape of the DNA "comet" tail and migration pattern gives an assessment of DNA damage. Whole blood was obtained by cardiac puncture, washed with cold phosphate buffered saline (PBS), and then centrifuged twice at 5,000 rpm for 5 min at 4°C. After removing the buffy coat, RBCs were washed twice in PBS. Cell mixture was suspended in Comet LMAgarose (1% low-temperature-melting agarose, Trevigen, Gaithersburg, MD, USA) at a ratio of 1:10 (v/v), 25 μl of the cell suspension was immediately placed on a CometSlideTM (Trevigen), slides were placed flat in a refrigerator at 4°C for 10 min, and then submerged in 23 ml of lysis solution (Trevigen) at 4°C for 60 min. Subsequently, slides were then maintained in an alkaline solution (>pH13) for 60 min at room temperature in the dark and washed two times in 1× TBE buffer (Tris-borate EDTA) for two times (5 min. each). Finally, slides were subjected to electrophoresis in 1× TBE buffer at 25 V for 10 min, stained with SYBR Green (Trevigen), and analysed under microscopy (IX-70, Olympus Co., Tokyo, Japan). Denatured cleaved DNA fragments migrated out of the cell under the stimulus of an electric potential, whereas the undamaged supercoiled DNA remained within the confines of the cell membrane when a current was applied. Parasites from α-TTP knockout with 18-29% of parasitaemia were recovered on day 21 after the infection. Parasites from C57BL/6J with 40-49% of parasitaemia were collected on day 12 after the infection. Four mice were used each experimental group.

### Detection of biomarker for oxidative stress with anti-8-hydroxy-2'-deoxyguanosine (8-OHdG) antibody

Collected blood from infected animals (n = 4) was mixed with an equivalent volume of PBS and then was centrifuged at 5,000 rpm for 5 min at 4°C, then the supernatant was removed (this step was repeated 3 times). The percentages of parasitaemia in α-TTP knockout and C57BL/6J mice were 27% and 35%, respectively. The pellet was resuspended with 0.1 ml of PBS and 0.1 ml of 3% foetal calf serum. The suspension was fixed with methanol for 10 min. Anti-8OHdG (2 ng/ml), a biomarker for the oxidative damage of DNA [17], monoclonal antibody (N45.1; MOG-20P; Japan Institute for the Control of Aging, Nikken SEIL Co. Ltd., Shizuoka, Japan) was labelled with Biotin-XX Mouse IgG1 according to the manufacturer's instructions (Z25052: ZenonTM Biotin-XX mouse IgG1, Molecular Probes, Eugene, USA), then this complex was incubated with the samples for 45 min at 37°C. The sample was washed twice with PBS for 5 min, and was incubated with streptavidine-Alexa Fluor 488 conjugate (Molecular probes) for 45 min at 37°C. The incubated sample was washed twice with PBS for 5 min. Parasite DNA was stained with propidium iodide (P1304 MP; Molecular Probes) containing RNase A (10109142001; Roche Applied Science, Mannheim, Germany) for 10 min at 37°C. After washing with PBS, the sample was treated with 1% n-propyl gallate (102747; MP Biomedicals, Irvine, CA USA), an anti-oxidant, and observed with a confocal laser microscope (DMRB/E, TCS NT; Leica Microsystems, Wetzlar, Hessen, Germany).

### Quantification of anti-oxidative stress enzymes of *P. berghei *NK65

The mRNA expression of anti-oxidative stress enzymes of *P. berghei *NK65, such as glutaredoxin (Grx), γ-glutamil transferase (γ-GCS), 2-Cys peroxiredoxin (2-Cys Prx) and thioredoxin reductase (TrxR), was monitored by a real time quantitative PCR carried out with specific double labelled probes in the ABI PRISM 7900 HT Sequence Detection System (Applied Biosystems, CA, USA). RBCs from infected and uninfected mice were separated from whole blood cells by using a gradient reagent (Histopaque-1077, Sigma, MO, USA). The sample was washed with cold PBS followed by centrifugation at 5,000 rpm for 10 min at 4°C in order to remove the residual buffy coat. This step was repeated twice. Then, the separated RBCs were treated with 0.15% saponin (Sigma-Aldrich, St. Louis, USA) and then were centrifuged and washed with cold PBS twice. The total RNA of parasites was extracted from the pellet by using TRI reagent (Sigma-Aldrich, St. Louis, USA) according to the manufacturer's instructions, and successively treated with Turbo DNA-freeTM reagent (Ambion, Texas, USA). The total RNA concentration was adjusted to 50 ng/μl. The mRNA amplification reaction (20 μl) consisted of 10 μl of 2× master mix without UNG, 0.5 μl of 40× multiscribe and RNase inhibitor mix, 1.8 μl of 10 μM of each forward and reverse primers, 0.8 μl of 5 μM TaqMan Probe, 4 μl of total RNA template (50 ng/μl) and 1.1 μl of RNase free double distilled water. The real time quantitative PCR first stage conditions were: 48°C for 30 min and the second stage of 45 reaction cycles with the following conditions: 95°C for 10 min, 95°C for 15 sec and 60°C for 1 min. The primers and probes used are shown in Table 1. Standard amplification curves were obtained by serial dilution of the total RNA of parasites. *Plasmodium berghei *18SrRNA was used as an internal control. Six mice were used for each experimental group.

**Table 1:** Primer and Probe sequences used in real--time quantitative PCR

Gene	Primer/probe
**18s rRNA**	5'-CGATGTGTGTCTAACACAAGGAAGT-3'F
	5'-CATAGGCTTTAACACCTAAGCACAG-3'R
	5'-FAM-TATGTAAAACGAGTGTTAAAT-MGB-3'
	
**2-Cys Prx**	5'-AAACACCATTGTCACAAGGAGGTA-3'F
	5'-ACAAACGCTCTTAATGCTACATTTC-3'R
	5'-FAM-AAGCATACTTTGATATCCG-MGB-3'
	
**g-GCS**	5'-AATGAGTATGTGCTGCCAACAAAT-3'F
	5'-AAGAATCCACCTAAATATGGTGTACATG-3'R
	5'-FAM-AGCTAGCTGTAATTGC-MGB-3'
	
**Grx**	5'-GGGAAAAGTTCCGTACCAAGAAT-3'F
	5'-TTCCAATGTCTGGAGTCTCTCTGA-3'R
	5'-FAM-ACCAGTCATCACATCCGCCGACATT-MGB-3'
	
**TrxR**	5'-AGTCACTCAAGGAATGGGATTGG-3'F
	5'-TGCATAAGACAAACCCGATGATAA-3'
	5'-FAM-TTCATCCAACAGATGCAG-MGB-3'

### Statistical analysis

Statistical analysis was performed using one-way variance analysis (S-plus6 software for windows) (Insightful corporation, Seattle Washington USA). Data are expressed as means of the standard error of the mean (SEM). P values less than 0.05 were considered to be significant. Survival rate % analysis was performed using the Kaplan-Meier method.

## Results

### Resistance of α-TTP knockout mice to malaria infection

**Figure 1:**
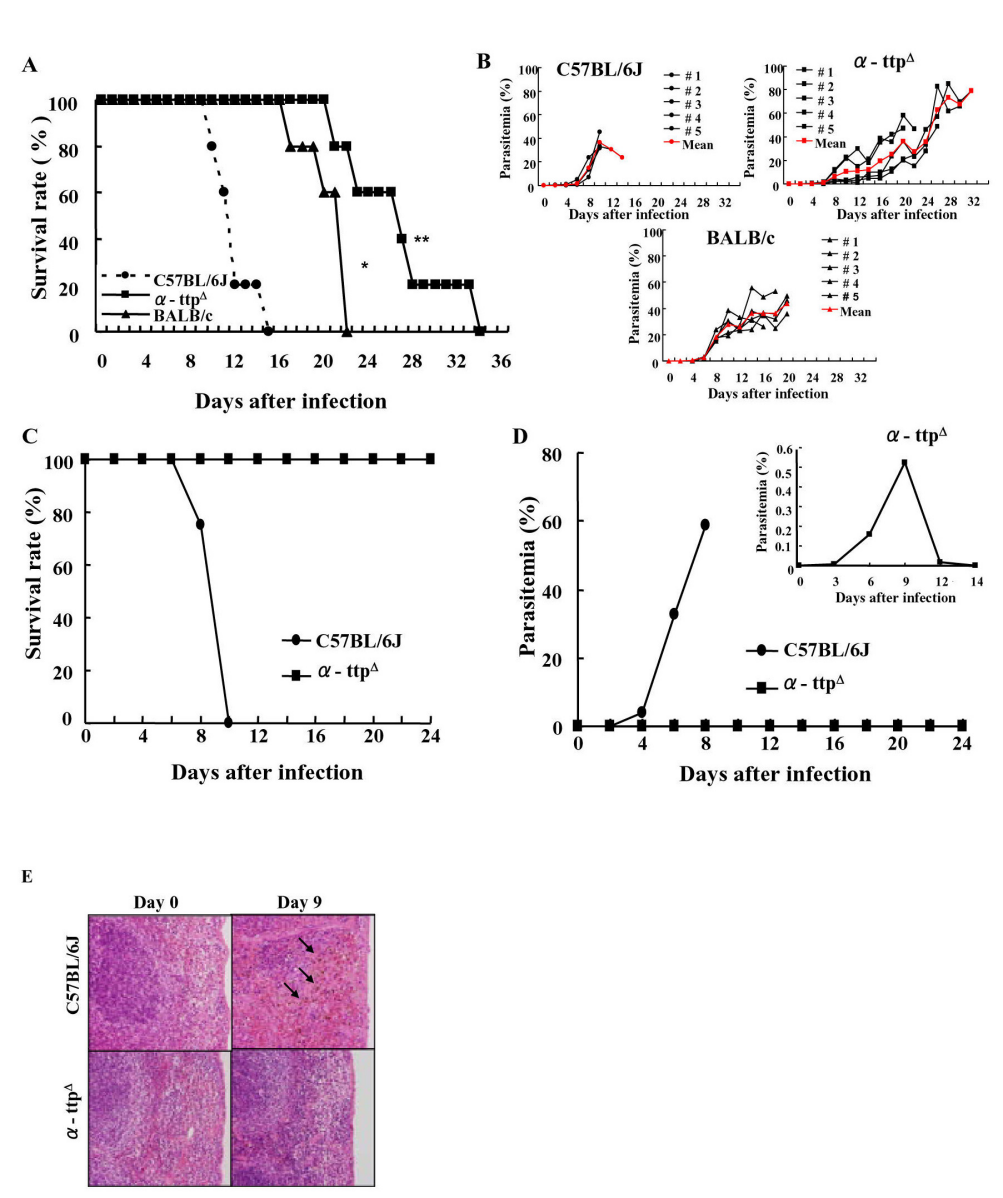
**Parasite proliferation inhibition in α-TTP knockout mice**. (A) Survival rates of α-TTP knockout mice (α-ttp^Δ^) infected with *P. berghei *NK65 were significantly longer than that of BALB/c and C57BL/6J mice (p < 0.01). (B) Percentages of parasitaemia for individual animals of each genotype. (C) Percentage of parasitaemia of α-TTP knockout mice infected with *P. yoelii*. (D) All α-TTP knockout mice infected with *P. yoelli *17XL survived with lower parasitaemia. The inset graph refers to a different scale of parasitaemia. (E) Malaria pigment (arrows) seen in the spleen of C57BL/6J mice but not in α-TTP knockout mice on day 9 after infection with *P. berghei *NK65 (n = 6). *: p < 0.05 C57BL/6J vs BALB/c; **: p < 0.01 C57BL/6J vs α-ttp^Δ ^in panel A.

When α-TTP knockout mice were infected with a lethal dose of *P. berghei *NK65 IRBCs, their survival was significantly extended compared to wild type (C57BL/6J) mice (p < 0.01) (Figure 1a). Furthermore, knockout mice harboured low parasite loads during the initial peak of parasitaemia, around day 10 post infection, when most wild type mice were dying (around day 12 post infection) (Figure 1b). Survival was longer and parasitaemia was lower in the α-TTP knockout than *P. berghei *NK65 resistant BALB/c and wild type mice. Lastly, inhibition of α-TTP did not trigger the development of anaemia during the acute phase of the infection; the total number of RBCs in knockout mice was 764 ± 47 × 10^4^/ul, comparable to wild type mice (871 ± 62 × 10^4^/ul). The effect of the α-TTP gene disruption was much more remarkable for a *P. yoelii *17XL infection that killed nearly all wild type mice by day 8; all knockout mice survived (Figure 1c). Furthermore, while some α-TTP knockout mice exhibited a trace level of parasitaemia at an early phase of infection, the IRBCs subsequently disappeared from circulation (Figure 1d). Taken together, it appears that knockout of *α-TTP *leads to inhibition of parasite development and subsequent longevity of the animals in the face of infection.

The spleen plays an important role during malarial infection removing uninfected and infected red blood cells from circulation. Histological analysis of spleens revealed the presence of haemozoin, a toxic haem product derived from the haemoglobin digestion thought to be an indication of macrophage phagocytic activity and parasite maturation [18], in the red pulp was visible by day 6 that increased in intensity by day 9 in wild type animals (Figure 1e). In addition, during the acute phase of the infection an increase in mature RBCs could be found indicating erythropoietic activity might be diminished in the wild type mice, a condition that could lead to anaemia. In contrast to wild type animals, α-TTP knockout mice displayed very little pigmentation or increase in mature RBCs even by day 9 indicative of delayed parasite maturation in these mice. In addition, mature RBCs and erythroblasts were intermixed in the red pulp of the spleen in α-TTP knockout mice indicating that red blood cell production is not affected in the infected mice early after infection. Moreover, the mRNA expression of erythropoietin receptor (EPOR) in bone marrow was significantly decreased in both genotypes on day three post infection, however the expression of EPOR was significantly increased in liver and spleen suggesting such organs compensate bone marrow function in order to avoid anaemia during the acute phase of the infection (manuscript under submission). These results indicate that α-TTP knockout animals are displaying no signs of infection even at a time when most wild type animals are dying.

**Figure 2:**
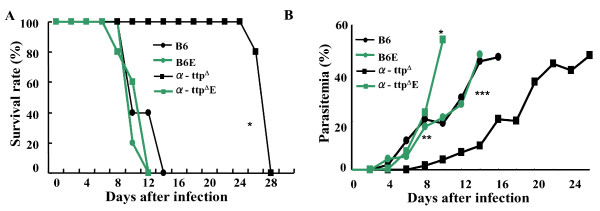
**Vitamin E supplementation reversed α-TTP knockout conferred resistance**. (A) Survival rate of α-TTP knockout mice fed with vitamin E supplementary diet (α-ttp^Δ^E) and infected with *P. berghei *NK65 was similar to the C57BL/6J mice fed to a normal diet (B6) or C57BL/6J mice fed to a supplemented diet of vitamin E (B6E). (B) Percentages of parasitaemia of α-TTP knockout mice fed with vitamin E supplementary diet was similar to the C57BL/6J mice. (n = 6) *: p < 0.05 B6, B6E, α-ttp^Δ^E vs α-ttp^Δ ^in panel A. In panel B *: p < 0.01 α-ttp^Δ ^vs α-ttp^Δ^E; ** p < 0.01 α-ttp^Δ ^vs B6E on day 10 post infection; ***: p < 0.01 α-ttp^Δ ^vs B6, B6E and α-ttp^Δ^E on day 14 post infection.

Plasma concentrations of vitamin E in the knockout and wild type mice fed to a normal diet were 0.02 ± 0.01 μM and 0.98 ± 0.12 μM, meanwhile, concentrations of vitamin E with the supplemented diet was 1.18 ± 0.08 μM and 3.3 ± 0.2 μM, respectively. Then, to analyse whether restoration of circulating vitamin E concentration in α-TTP knockout mice could lead to malaria susceptibility, mice were fed a diet supplemented with α-tocopherol (600 mg/kg diet) ten days before infection with *P. berghei *NK65. Survival curves and parasitaemia kinetics of knockout mice fed an excess amount of α-tocopherol-supplemented diet, were similar to wild type (Figures 2a-b). These results demonstrate that α-TTP knockout mice acquired resistance to malaria infection by vitamin E deficiency, not by *α-TTP *gene disruption itself.

### DNA damage of Plasmodium in α-TTP knockout mice

**Figure 3:**
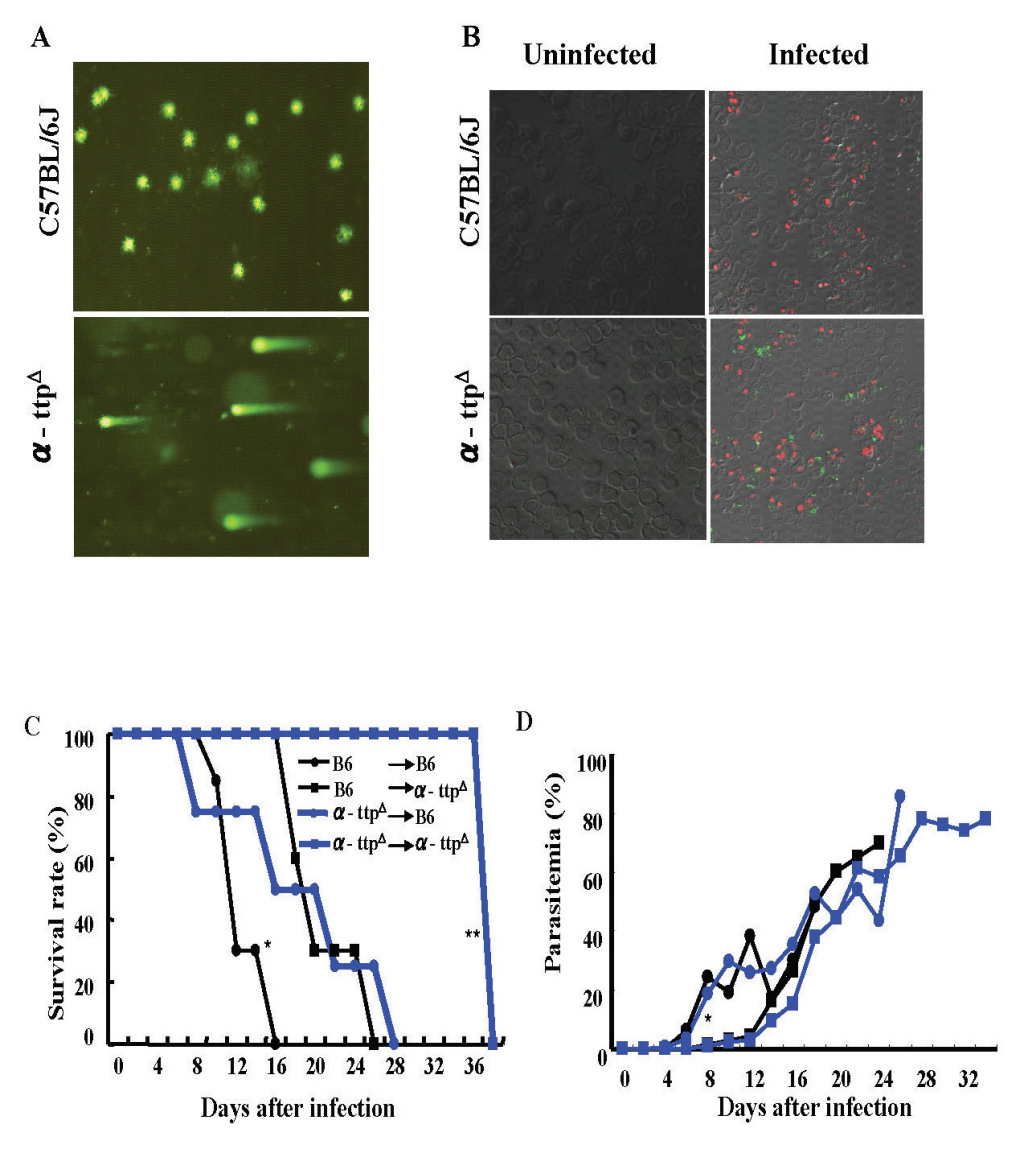
**Vitamin E deficiency leads to *P. berghei *NK65 DNA damage**. (A) Comet assay, indicating severe DNA damage, on the parasites recovered from α-TTP knockout or C57BL/6J mice. (B) Malarial parasites from α-TTP knockout and C57BL/6J mice stained with anti 8-OHdG (green), a biomarker of oxidative DNA damage. The DNA of the parasites was stained with propidium iodide (red) (n = 4). (C) C57BL/6J mice infected with parasites recovered from the α-TTP knockout (α-ttp^Δ ^→ B6) or C57BL/6J mice (B6 → B6). α-TTP knockout mice infected with parasites recovered from α-TTP knockout mice (α-ttp^Δ ^→ α-ttp^Δ^) or C57BL/6J mice (B6 → α-ttp^Δ^) (n = 6). *: p < 0.05 B6 → B6 vs α-ttp^Δ ^→ B6; **: p < 0.01 B6 → α-ttp^Δ ^vs α-ttp^Δ ^→ α-ttp^Δ^in panel C. In panel D *: p < 0.05 B6 → B6, α-ttp^Δ ^→ B6 vs B6 → α-ttp^Δ^, α-ttp^Δ ^→ α-ttp^Δ^.

Since decreased vitamin E concentrations likely lead to an environment of high oxidative stress, DNA damage in these parasites was assessed. A comet assay evaluates the shape of the DNA "comet" tail and migration pattern as an indication of DNA damage [16] and revealed that the parasite-infected RBCs in α-TTP knockout mice had severe DNA damage (Figure 3a). This was in contrast to infected RBCs from wild type mice where parasites displayed no such comet tails. Immunofluorescence staining of anti-8 hydroxy-2-deoxyguanosine (8-OHdG), a biomarker for oxidative DNA damage [17], confirmed oxidative DNA damage to parasites recovered from α-TTP knockout mice (Figure 3b). Taken together it appears that loss of α-TTP leads to DNA damage of hosted *Plasmodium *parasites, presumably due to consequent decreased vitamin E concentration and its associated free radical scavenging activity.

To elucidate whether the virulence of parasites in α-TTP knockout mice might be affected during infection due to parasite DNA damage, mice were inoculated with *P. berghei *NK65 that had been recovered from previously infected α-TTP knockout or wild type mice 9 days post infection. Tellingly, both the α-TTP knockout and wild type mice infected with parasites recovered from the α-TTP knockout mice survived significantly longer than the mice infected with parasites recovered from wild type mice (Figure 3c). Surprisingly, despite increased longevity, parasitaemia kinetics in wild type mice and knockout mice were similar irrespective of the source of the parasites (Figure 3d). These results suggest that the virulence of the parasites existing in α-TTP knockout mice had decreased during their time within that host.

### Expression of anti-oxidative enzymes in infected parasites

**Figure 4:**
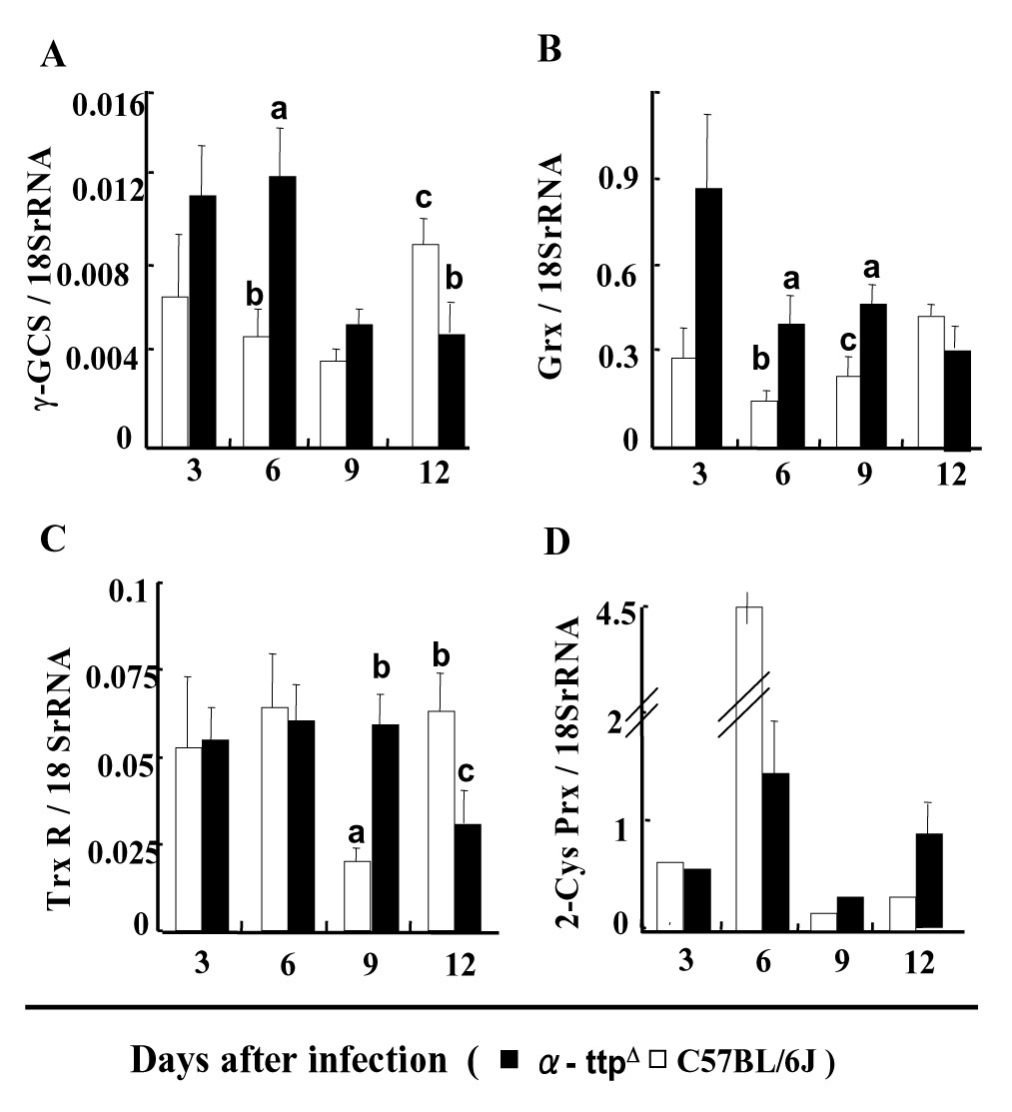
**Expression of anti-oxidative enzymes in parasites**. mRNA expression of glutaredoxin (Grx), γ-glutamylcysteine synthetase (γ-GCS), 2-Cys peroxiredoxins (Prx) and thioredoxin reductase (TrxR) of infected parasites in the C57BL/6J (white bar) and α-TTP knockout mice (black bar). Bars represents ± SEM; Bars not sharing common letters are significantly different (*p *< 0.05) by Scheffe multiple comparison test. (n = 6).

After infection with *P. berghei *NK65, the mRNA expression of anti-oxidative stress enzymes, such as glutaredoxin (Grx), γ-glutamil cysteine synthetase (γ-GCS), 2-Cys peroxiredoxin (2-Cys Prx) and thioredoxin reductase (TrxR), of the parasites was examined using real time quantitative PCR. Two key anti-oxidant genes that have been used frequently to monitor the status of the anti-oxidant machinery of parasites, such as *γ-GCS *and *Grx*, showed higher levels of expression at initial points in the knockout mice compared to the wild type mice (Figures 4a-b). Parasites from knockout mice showed steady levels of *TrxR *expression until day 12 post-infection when it dropped (Figure 4c). These levels were comparable to those of parasites from wild type hosts. However, *TrxR *expression dropped dramatically at day 9 before recovering at day 12 in the wild type mice. Despite the similar levels of expression in *TrxR *between knockout and wild type mice, it appears that parasites have activated anti-oxidant systems at the earliest time points of infection in the knockout mice. Increased transcription of anti-oxidant genes in infected α-TTP knockout mice was in contrast to infected wild type animals where only *2-Cys Prx *expression was increased (Figure 4d).

### Parasite invasion into RBCs

**Figure 5:**
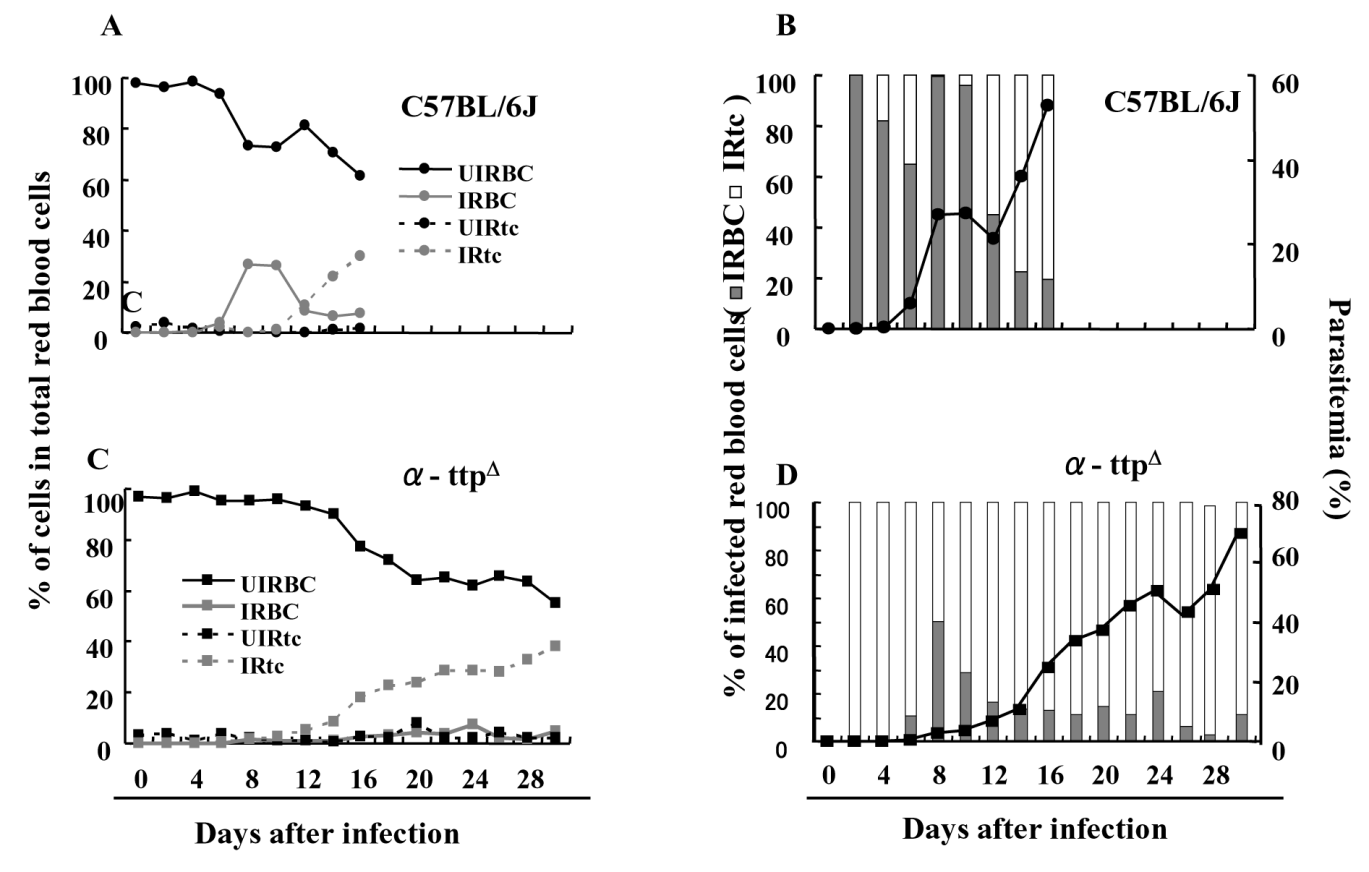
**Parasites infecting α-TTP knockout mice intend to invade immature RBCs**. Percentages of uninfected mature RBCs (UIRBC), infected mature RBCs (IRBC), uninfected reticulocytes (UIRtc), and infected reticulocytes (IRtc) from total RBCs of C57BL/6J in panel A and α-TTP knockout mice in panel C. Percentages of infected mature RBCs (gray bar) and infected reticulocytes (white bar) from total infected RBCs of C57BL/6J in panel B and α-TTP knockout mice in panel D (n = 6).

In the absence of infection no significant difference in the percentage of mature RBCs or immature RBCs (reticulocyte) within the total RBCs was observed between α-TTP knockout and wild type mice (Figures 5a-c). However, throughout infection the percentages of infected reticulocytes (IRtc) in the total infected RBCs were significantly higher (p < 0.05) in α-TTP knockout mice than wild type animals (Figures 5b-d). These results support the notion that parasites in knockout mice tend to invade newly produced cells rather than mature RBCs, likely to evade the increased oxidative stress derived from vitamin E deficiency. This is in contrast to parasites in wild type mice that preferentially invaded mature RBCs during the acute phase of infection, and afterwards displayed a tendency to invade immature RBCs, probably due to a decrease in the number of mature RBCs that were available (Figure 5b). Taken together it appears that a high oxidative stress environment of knockout mice RBC leads to *Plasmodium *parasites to a preferential invasion of immature RBCs.

### Effect of chloroquine administration coupled with α-TTP disruption

**Figure 6:**
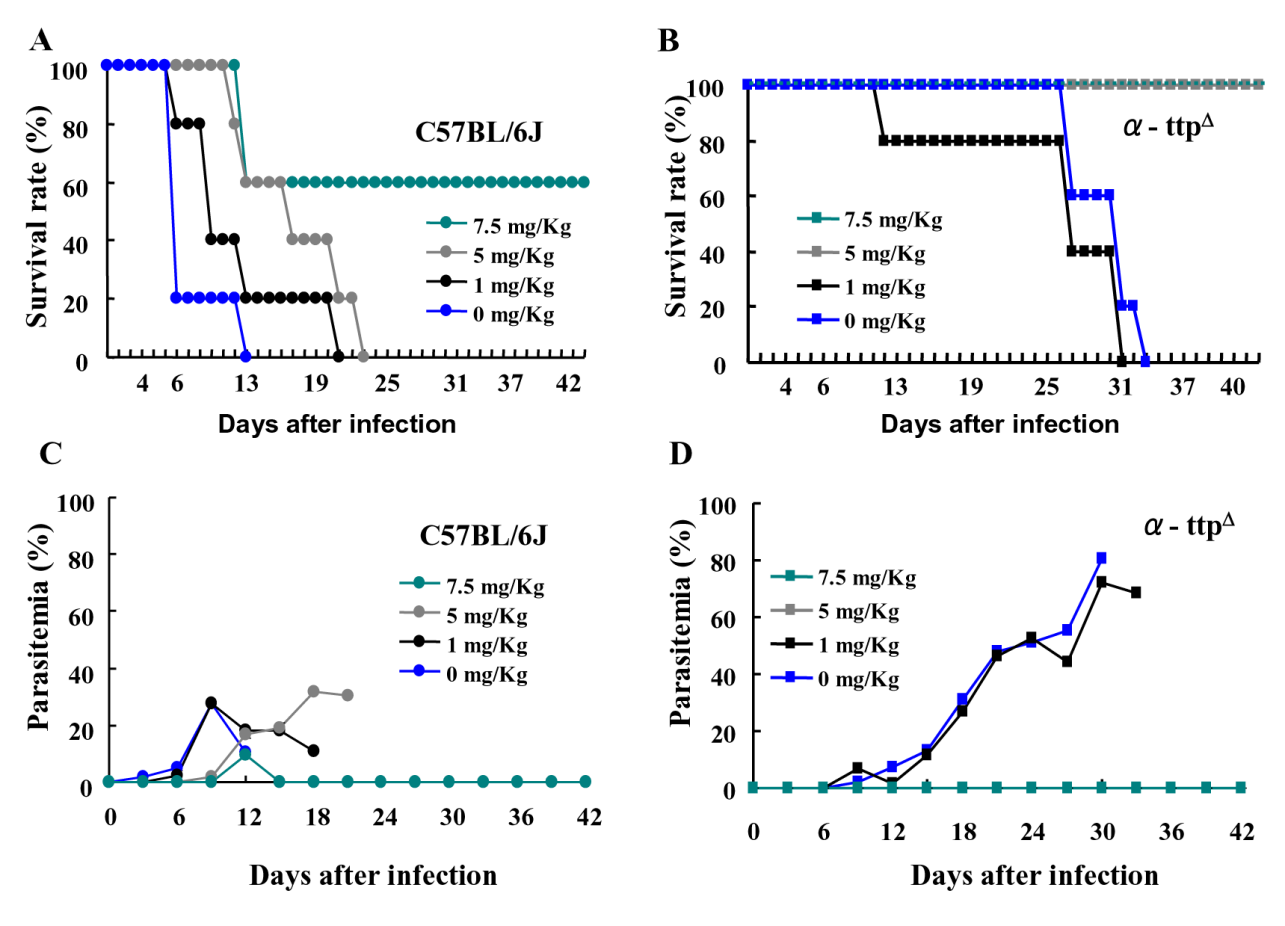
**Chloroquine administration in combination with α-TTP disruption inhibits parasite proliferation**. Mice were infected with *P. berghei *NK65 (4 × 10^5 ^IRBCs) and administered 0-7.5 mg/Kg of chloroquine on days 0, 1, and 2 after infection (n = 6).

The possibility of combining α-TTP inhibition with existing malaria treatment in the form CQ treatment was examined [19]. When wild type mice were treated with 5 mg/kg of CQ their survival to *P. berghei *NK65 infection was significantly improved (Figure 6a), however, parasitaemia continued to increase beginning at day 9 and mice succumbed eventually to malaria by day 23 (Figures 6a-b). In contrast, identical treatment of α-TTP knockout mice resulted in 100% survival. Strikingly, these mice displayed undetectable levels of parasitaemia (Figures 6c-d). These results indicate that a combination of *α-TTP *inhibition and CQ administration could potentially be used as a useful treatment for malarial infection.

## Discussion

Previous reports have indicated that reduction of host Vitamin E concentration through dietary restriction negatively impacted the development of *Plasmodium *and malaria development [11,12,20]. From 1954 to 1971, the notion that malnutrition was protective against malaria infection was promoted. This idea was strongly supported by epidemiological studies made from 1975 to 1980 by Murray *et al *[11]. In addition, animal studies appeared to support this idea. For example, Levander *et al *[21] demonstrated that vitamin E and selenium status have a profound impact on the ability of the host to resist acute infectious diseases. The relation between nutritionally-induced oxidative stress and malaria was firstly studied using the Chinese traditional anti-malarial drug, Qinghaosu. This compound is thought to destroy the parasite by generating oxy-free radicals. Therefore, the idea of dietary deprivation of vitamin E, a free radical scavenger, might be expected to enhance the anti-malarial activity of the drug [21]. In this study, rather than reduce vitamin E via diet, the α-TTP gene responsible for regulation of host vitamin E concentration was inhibited. Modification of host nutritional status through inhibition of α-TTP and subsequent lowering of vitamin E concentration conferred resistance to malarial infection (Figure 1).

Parasite proliferation was dramatically inhibited in the knockout mice infected with *P. yoelii *compared to the knockout mice infected with *P. berghei *NK65. The increased sensitivity to oxidative damage of *P. yoelii *compared to *P. berghei *has been demonstrated [22]. In these studies, mice infected with *P. yoelii *and treated with intravenous injection of H_2_O_2 _almost cleared parasites compared to those infected with *P. berghei *in which parasitaemia still increased. This phenomenon was due to an earlier and higher production of O_2 _in the splenic macrophages as compared with the mice infected with *P. berghei*. Moreover, mice infected with *P. yoelii *sporozoites showed a decrement in the cellular inflammatory response against infection [23]. Therefore, it is speculated that vitamin E deficiency enhances the susceptibility of *P. yoelii *to oxidative damage due to the exacerbative production of ROS from the immune response of the host and likely due to a decreased immune response. Moreover, α-TTP knockout mice exposed to 10-20 *P. berghei *ANKA infected mosquitoes displayed similar survival rates to the α-TTP knockout mice infected by IRBCs via intraperitoneal injection (Additional file 1). Thus, it seems that *α-TTP *gene disruption does not influence development of the sporozoite stage of parasites in the liver (pre-erythrocytic stage). It is well known that vitamin E, through its free radical scavenging properties, prevents damage of molecules such as DNA, lipids and proteins in the face of production and biotransformation of ROS in biological systems [24]. Consistent with the notion that malaria resistance in α-TTP knockout mice resulted from lost vitamin E scavenging activity, reduced *Plasmodium *proliferation was reversible upon vitamin E supplementation (Figure 2). The absence of vitamin E in the host cells would lead to an oxidative stressful environment that would require parasites to efficiently use their anti-oxidant systems for survival such as the thioredoxin system [25-27].

Furthermore, anti-oxidant activity in these parasites was raised and DNA damage was evident. After initial high levels of expression the transcription of *γ-GCS*, *Grx *and *TrxR *genes dropped in the knockout mice, while parasitaemia increased presumably as the parasites adapted to the oxidative environment [28]. This is in contrast to parasites hosted within wild type mice where the expression of *γ-GCS *and *Grx *increased over time, as haemoglobin consumption would be expected to occur and consequently create an oxidative environment [29] (Figures 3a-b). Taken together, it appears that inhibition of *α-TTP *gene activity leads to an inhospitable environment for parasites residing within the host. Since the nature of dietary fat alters the lipid composition of RBC membranes and malarial parasites can not biosynthesize their own fatty acids, likely, parasites and RBC membranes of mice fed with vitamin E deficient diets enriched with fish oil containing diets are highly susceptible to peroxidation due to a normal production of ROS [20]. In this study, knockout mice fed with normal diet of vitamin E did not show early after infection stressing that one of the reasons of inhibition of parasite proliferation in the knockout mice is clearly related to oxidative damage of the parasite. This suggests that knockout mice might possess additional mechanisms to keep normal levels of vitamin E in the RBC membranes [30]. It is impressive that α-TTP knockout mice survived with rising levels of parasitaemia at the later stage of the infection. Possible explanation might be that hosts surviving initial infection selected parasites less able to cause death to hosts. However, it is important to point out that whilst parasitaemia increases, anaemia status is more severe, suggesting that anaemia is a defense mechanism of the host for the prevention of parasite proliferation. Also, it can be speculated that parasites at this point are less virulent that the ones during the acute phase of the infection. Evidence for this notion comes from Comet assay revealing DNA damage to parasites hosted within the α-TTP knockout mice at the earliest points after infection. Furthermore, parasites recovered from these mice were less virulent than those recovered from the wild type mice; indeed, wild type mice infected with knockout recovered parasites survived significantly longer than those infected with wild type recovered parasites (Figures 3c-d). While these finding potentially explain the survival of knockout mice despite high levels of parasitaemia, they do not explain the observation of wild type mice surviving initial parasitaemia. This phenomenon, however, is certainly worth future study and may be used to gain insight into similar dynamics in human populations with malaria.

In this study, the mRNA expression of IL-10, INF-γ and TNF-α in liver, kidney, and spleen throughout infection was similar in the knockout mice as compared to the wild type mice suggesting that the inflammatory-immune response of the knockout mice is not altered by *α-TTP *disruption (manuscript in preparation). It has been suggested that the acquired immune response of the host likely play an important role in the parasite elimination [20]. When α-TTP knockout mice were infected with *P. yoelii *and re-infected with *P. berghei *ANKA on day 14 post-infection, 80% of the knockout mice were able to survive showing a very low parasitaemia. In contrast, 20% of the knockout mice infected with *P. berghei *ANKA or *P. yoelii *and *P. berghei *ANKA simultaneously were able to survive, indicating that knockout mice might be able to generate an acquired immunity or produce malarial antibodies against parasites. In this study parasite infecting the knockout mice were able to proliferate from day 15 after infection suggesting that either parasites might develop a resistance characteristic against malarial antibody or host protective immune response is impaired in these mice due to a mechanism of adaptation between parasite and mice. The mRNA expression of *IL-10, INF-γ*, and *TNF *in liver, kidney, and spleen throughout infection was similar in the knockout mice as compared to the wild type mice.

With the finding that inhibition of α-TTP leads to malaria resistance, potential exploitation as a novel therapeutic strategy becomes possible. Interestingly, inhibition of *α-TTP *did not trigger development of anaemia as indicated by the finding that no significant differences in RBC/reticulocyte ratio or total numbers of RBCs could be seen between the α-TTP knockout and wild type mice in uninfected mice (Figure 5a). Moreover, RBCs from the α-TTP knockout were normal in appearance and quality, the maximum and minimum resistance to osmotic stress at different concentrations of NaCl were the same (Additional file 2). Lastly, the concentration of reduced glutathione, an indicator of oxidative status, in the liver unchanged 19.3 ± 1.28 and 17.0 ± 1.20 for the C57BL/6J and α-TTP knockout mice, respectively (Additional file 3). Either α-TTP knockout mice absorb vitamin E from their diet to maintain transiently acceptable levels of vitamin E within the RBCs, as has been demonstrated in patients with familial isolated vitamin E deficiency [30], or other anti-oxidants such as ascorbate and β-carotene compensate for missing vitamin E in RBC membranes of α-TTP knockout mice [24]. Based on these observations it appears that α-TTP gene inhibition is a potentially valid therapeutic strategy.

Another prerequisite of *α-TTP *gene disruption as a potential therapeutic strategy lies in the ability to chemically inhibit the protein. Fortunately, in humans, a binding pocket in α-TTP, specific for α-tocopherol, has been revealed by crystallography and is considered to be responsible for the homeostasis of vitamin E [31]. Disruption of α-TTP inhibits α-tocopherol transfer from the liver and thus, would serve as an ideal target in drug design.

One of the most exciting findings from this study lies in the use of combinations of CQ [19], a classic anti-malarial drug, and gene disruption; levels of CQ found to leave parasites remaining in wild type mice removed all traces of the parasite from α-TTP knockout mice. By using such a combination, levels of CQ known to be toxic to patients could be avoided. Furthermore, drug resistance of *Plasmodium*, a serious problem in many parts of the world, could be avoided in a manner similar to avoidance of HIV drug resistant strains through combinations of drugs [32]. In terms of genetics, this duel approach is similar to synthetic lethality whereby simultaneous disruption of two genes results in a synergistic effect that is greater than either single gene disruption [32-34]. The finding that the presence of CQ is able to impair the intracellular alpha tocopherol transport suggests that CQ treatment and disruption of *α-TTP *likely are impinging on the same cellular process and therefore prone to such a synthetic therapeutic approach [35]. This synthetic lethal approach has been used recently for drug discovery [36] and has begun to be exploited in cancer research [37,38]. Lastly, it is possible that this type of strategy might be extended to other parasites as α-TTP gene disruption was found to be protective against a variety of parasites [39].

## Conclusion

Inhibition of α-TTP activity in host leads to a resistance characteristic against malaria infection due to parasite's DNA oxidative damage. Therefore, α-TTP inhibition in host might be a new strategy for its prevention and control. Moreover, a combined strategy of α-TTP inhibition and chloroquine treatment might be effective against drug resistant parasites.

## Competing interests

The authors declare that they have no competing interests.

## Authors' contributions

MSH designed, conducted, analysed the data and wrote the manuscript, YYU conducted the experiments, CI conducted the experiments, MC conducted the experiments, KI conducted the experiments, MS advised for the vitamin E concentration measurements, SF conducted the experiments, NY conducted the experiments, MT analysed the data, XX analysed the data, HA analysed the data, HS conceived and designed the study and contributed to the preparation of the manuscript. All authors have read and approved the final manuscript.

## Supplementary Material

Additional file 1 **Survival rates of α-TTP knockout mice infected with *P. berghei *ANKA via mosquitoes or IRBCs**. α-TTP knockout mice exposed to 10-20 P. berghei ANKA infected mosquitoes displayed similar survival rates to the α-TTP knockout mice infected by IRBCs via intraperitoneal injection. Click here for file

Additional file 2 **The maximum and minimum resistance to osmotic stress at different concentrations of NaCl in RBCs from the α-TTP knockout mice**. The maximum and minimum resistance to osmotic stress at different concentrations of NaCl in RBCs from the α-TTP knockout mice were similar to those from wild type mice. Click here for file

Additional file 3 **Reduced glutathione concentrations of the liver in α-TTP knockout and wild type mice**. Reduced glutathione concentrations of the liver were similar in α-TTP knockout and wild type mice. Click here for file
