# Supplementary figures and images for: Alpha-tocopherol transfer protein disruption confers resistance to malarial infection in mice
Source: Malar J. 2010 Apr 19;9:101. doi: 10.1186/1475-2875-9-101 (PMC2862040; doi:10.1186/1475-2875-9-101)

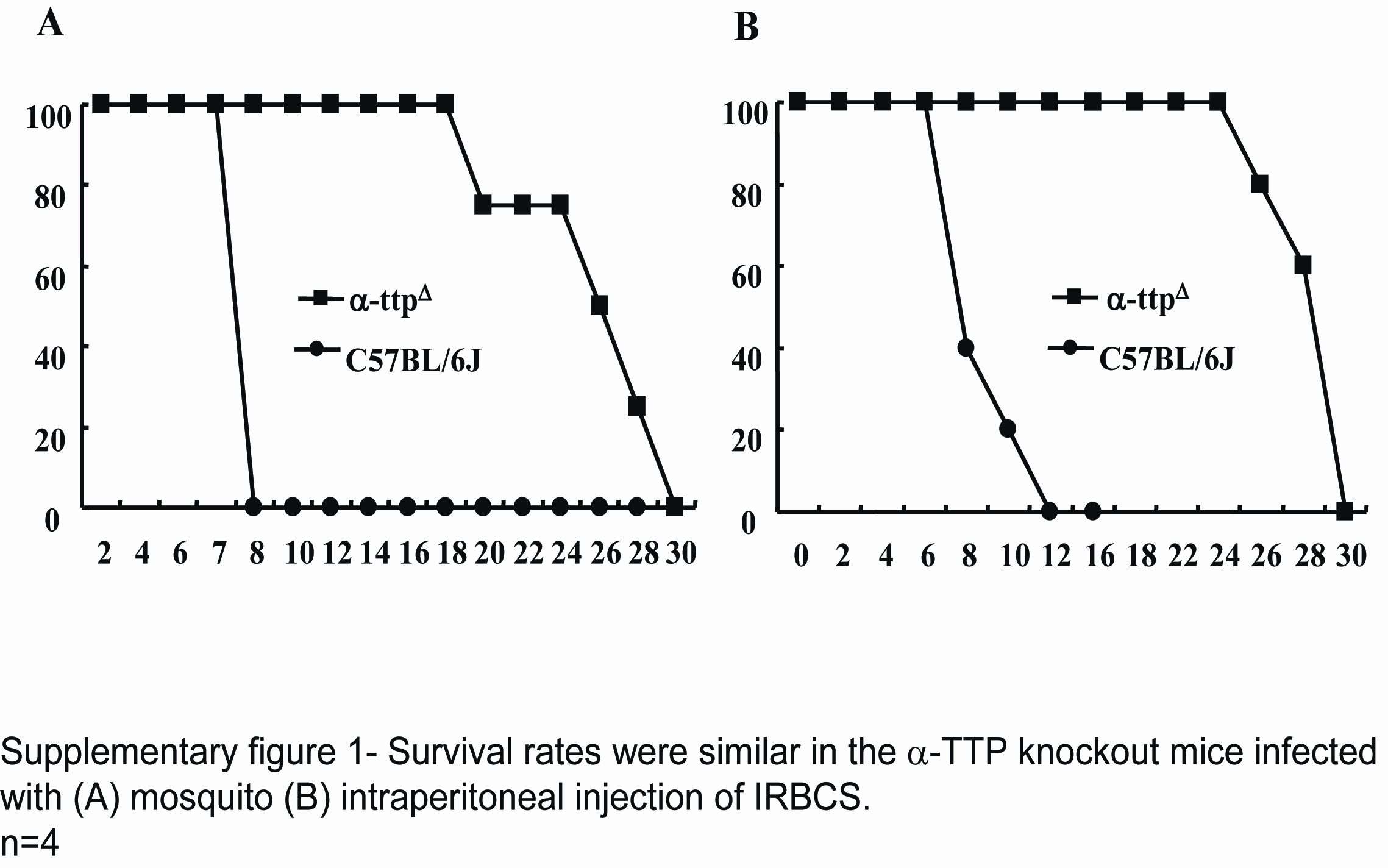

Supplement: Additional file 1 — Survival rates of α-TTP knockout mice infected with P. berghei ANKA via mosquitoes or IRBCs. α-TTP knockout mice exposed to 10-20 P. berghei ANKA infected mosquitoes displayed similar survival rates to the α-TTP knockout mice infected by IRBCs via intraperitoneal injection. [file 1475-2875-9-101-S1.TIFF]

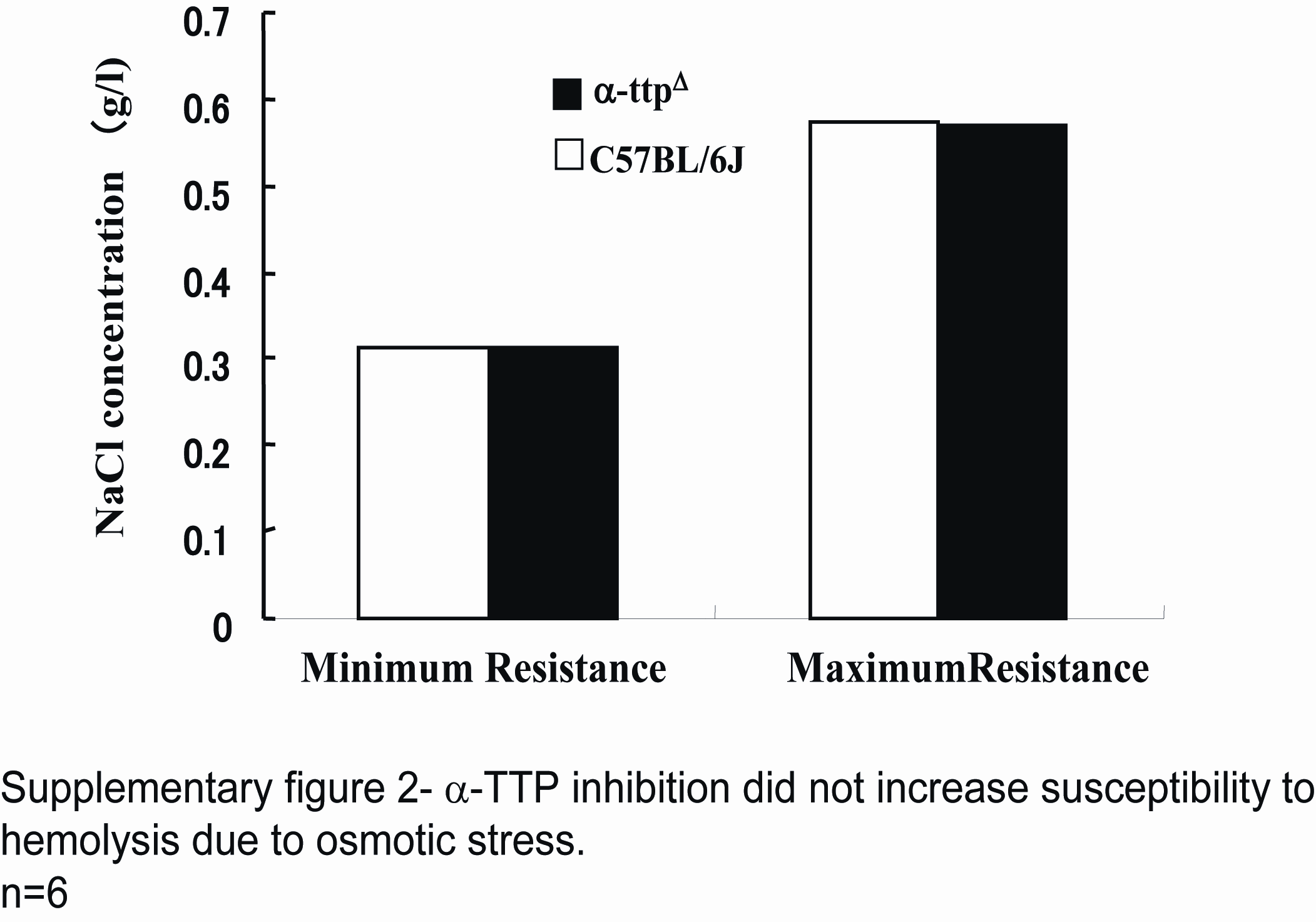

Supplement: Additional file 2 — The maximum and minimum resistance to osmotic stress at different concentrations of NaCl in RBCs from the α-TTP knockout mice. The maximum and minimum resistance to osmotic stress at different concentrations of NaCl in RBCs from the α-TTP knockout mice were similar to those from wild type mice. [file 1475-2875-9-101-S2.TIFF]

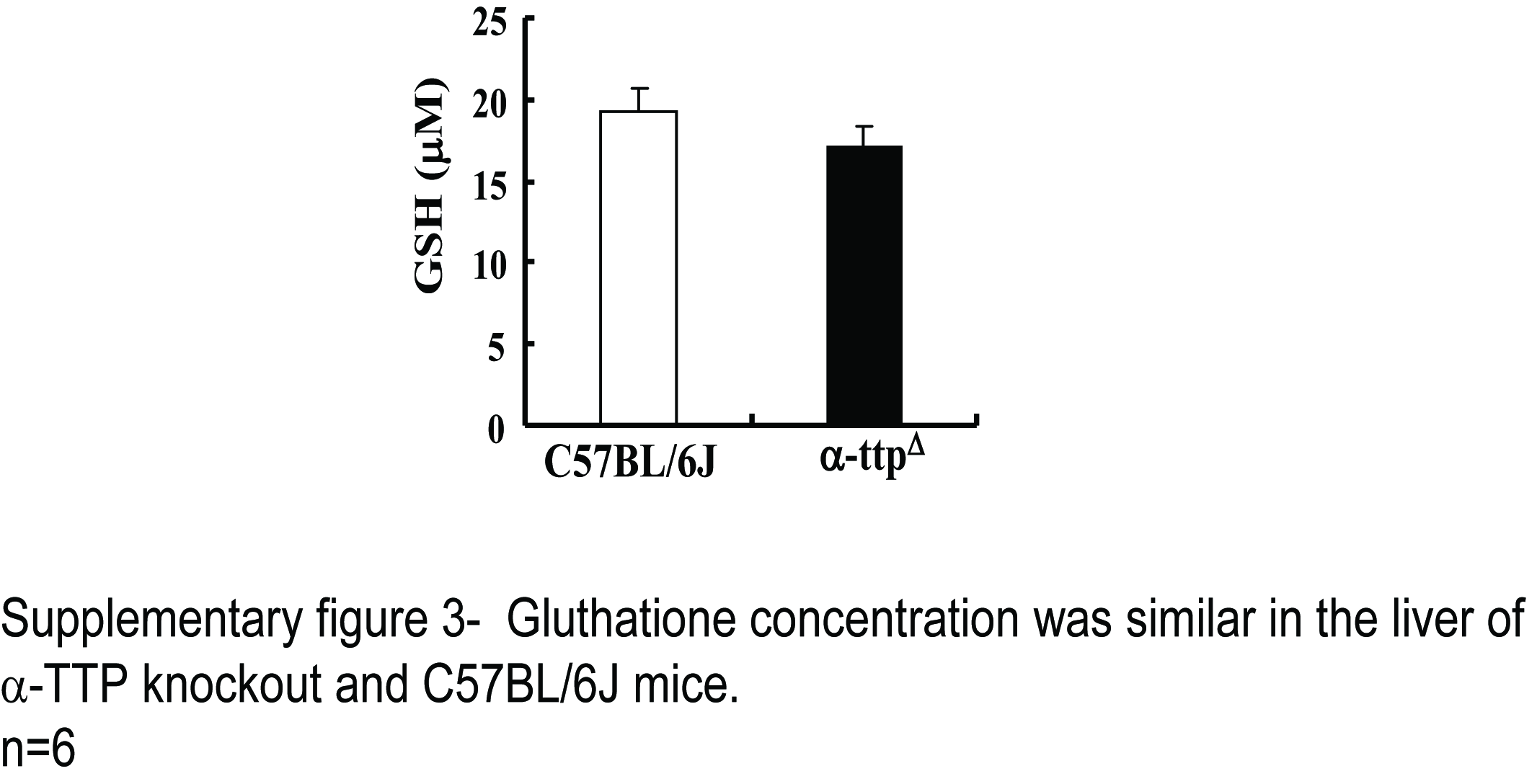

Supplement: Additional file 3 — Reduced glutathione concentrations of the liver in α-TTP knockout and wild type mice. Reduced glutathione concentrations of the liver were similar in α-TTP knockout and wild type mice. [file 1475-2875-9-101-S3.TIFF]
